# Quantitative magnetooptical analysis using indicator films for the detection of magnetic field distributions, temperature, and electrical currents

**DOI:** 10.1038/s41598-024-74684-y

**Published:** 2024-10-26

**Authors:** Michael P. Path, Jeffrey McCord

**Affiliations:** 1 Nanoscale Magnetic Materials - Magnetic Domains, Department of Materials Science, Faculty of Engineering, Kiel University, 24143 Kiel, Germany; 2 Kiel Nano, Surface and Interface Science (KiNSIS), Kiel University, 24118 Kiel, Germany

**Keywords:** Magnetooptics, Temperature sensing, Magnetic field sensing, MOIF, Magnetooptical microscopy, Materials science, Materials for devices, Materials for optics, Techniques and instrumentation, Optics and photonics, Applied optics, Optical techniques, Engineering, Electrical and electronic engineering

## Abstract

The accurate characterization of local magnetic fields and temperature is vital for the design of electronic systems. To meet this imperative, we present a novel non-contact approach for simultaneous quantitative magnetic field imaging and temperature sensing using magnetooptics and a bismuth-doped yttrium iron garnet film with out-of-plane anisotropy. For the direct signal quantification, a Stokes polarization camera is employed in a conventional magnetooptical microscope. The magnetization in the garnet is modulated with an external magnetic field to continuously image the Faraday rotation at four distinct points along the saturating magnetization loop. The method enables sensing of the magnetooptical signal in saturation, the magnetooptical susceptibility, the temperature, and self-calibrated driftfree imaging of the out-of-plane magnetic field component. A spatial resolution of magnetic field in the micrometer range with millisecond exposure time is demonstrated. The method is verified by analyzing the stray magnetic field distribution of electrical current in a wire simultaneously to the Joule heating induced by the applied current.

**Supplementary Information:**

The online version contains supplementary material available at 10.1038/s41598-024-74684-y.

## Introduction

**Fig. 1:**
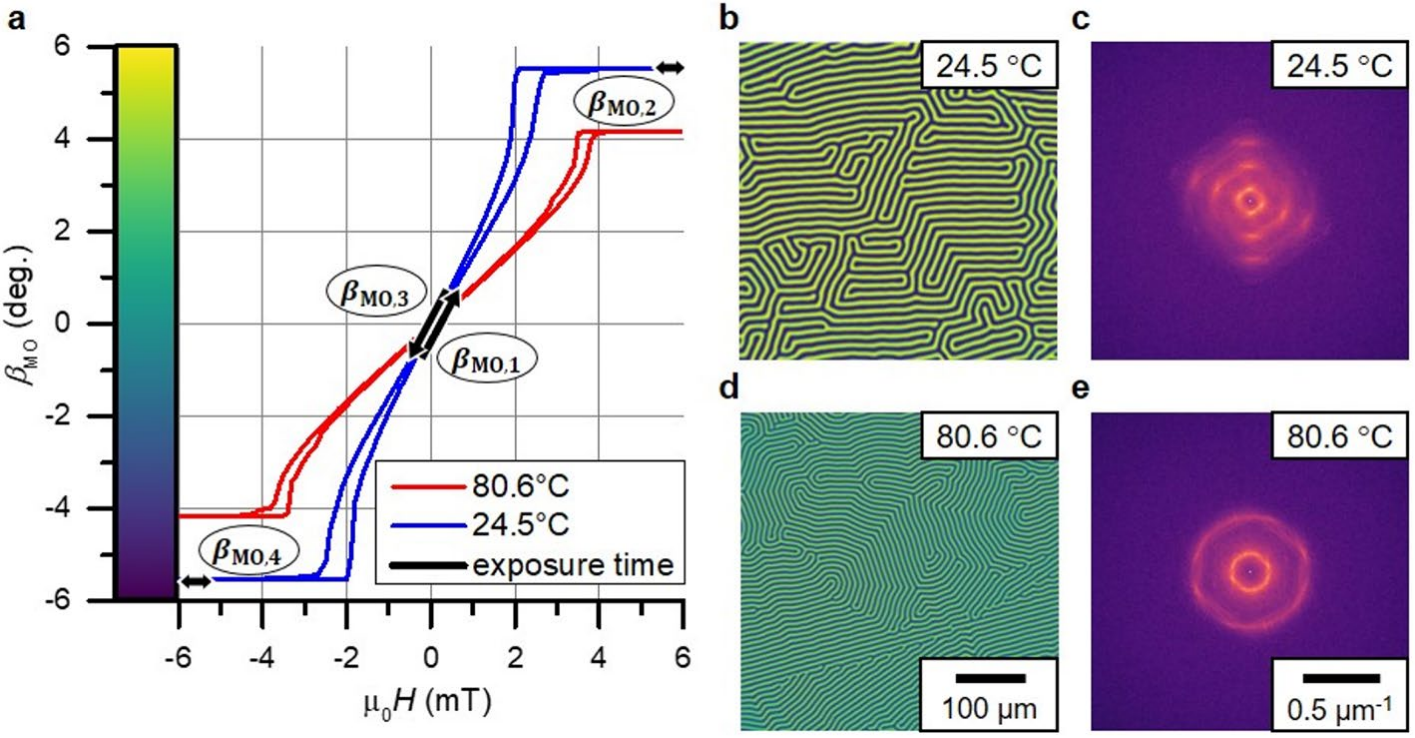
Temperature dependence of magnetization loop shape and magnetic domain structure. (**a**) Quantitative magnetooptical magnetization loops of the YIG sensor with OOP anisotropy at 24.5 °C and 80.6 °C. The parts highlighted by arrows correspond to the swept field during the exposure time (3.3 ms) of four chronologically numbered images taken during an external sinusoidal field modulation (period 71.4 ms) for one combined measurement. The difference of ${\beta}_{\text{M}\text{O},2}$ and ${\beta}_{\text{M}\text{O},4}$ corresponds to the maximal Faraday rotation in saturation ${\beta}_{\text{M}\text{O},\text{s}\text{a}\text{t}}$, while the average of ${\beta}_{\text{M}\text{O},1}$ and ${\beta}_{\text{M}\text{O},3}$ scales with a possible superimposing DC-field. Quantitative domain images of the demagnetized state along with the corresponding Fourier transformed images at (**b**), (**c**) 24.5 °C and (**d**), (**e**) 80.6 °C show the temperature dependence of the domain period of the labyrinth structure and the signal difference between the magnetic domains.

Quantifiable measurements of electrical currents, magnetic fields, and temperatures remain of ongoing interest in both laboratory and industrial settings. The possible applications range from power electronics^1^ and integrated circuits^2^ to the development of superconducting circuits^3^. Via localization of thermal sources and currents, potential defects like shorts-circuits can be detected. This is relevant both in quality control as well as testing during development to prevent causes of failure. A magnetooptical detection enables a galvanic separation, ensuring safety and preventing anomalous currents from potentially compromising the results^2,4^, as well as a fast read out over a large area^5^. Alternative methods to measure currents indirectly via stray magnetic fields with various resolutions and sensitivities include SQUID (superconducting quantum interference devices), magnetoelectric sensors^6^, scanning Hall probe microscopy^7^, quantitative magnetic force microscopy^8^, nitrogen vacancies^9^ and magnetoresistive devices using anisotropic (AMR) or giant magnetoresistance (GMR) sensors^2^. Ferrimagnetic magnetooptical indicator films (MOIF) are a well-known sensor material for imaging stray magnetic fields of direct currents^10^. Most research using magnetooptics for quantified out-of-plane (OOP) magnetic field imaging up to now focuses on MOIFs with in-plane (IP) anisotropy^10–15^ as they show a linear OOP susceptibility for a large range of magnetic fields. In addition, they exhibit almost no hysteresis along this direction, as there is no domain wall movement involved. In contrast, less research has been put into quantitative imaging with indicator films with OOP anisotropy^13,16^. They are used to image and quantify stray fields of e.g. electrical steel^17,18^. Typically, garnet films with OOP anisotropy exhibit a non-reproducible labyrinth structure of their magnetic domains during a magnetization loop with applied fields perpendicular to the sample plane. As depicted in Fig. 1, both hysteresis and domain structure depend on temperature. MOIFs with OOP anisotropy typically exhibit an order of magnitude higher susceptibility than their IP counterparts, indicating their potential as highly sensitive sensors. On the other hand, relying on spatially and temporally varying magnetic domain characteristics for sensing is a significant challenge. In addition, MOIFs have been demonstrated as suitable temperature sensors using the temperature dependency or pyro-magnetism of susceptibility^19^, or saturation magnetization^20^ along the plane normal. An alternative method for the simultaneous measurement of temperature and magnetic fields with an IP-MOIF utilizes the thermochromism of the sensor^21^. The temperature is measured in the optical microscope using the change of the field independent absorption of the overall light intensity. Other comparable optical temperature measurement techniques include infrared thermal microscopy or Raman thermometry^21^.

**Fig. 2:**
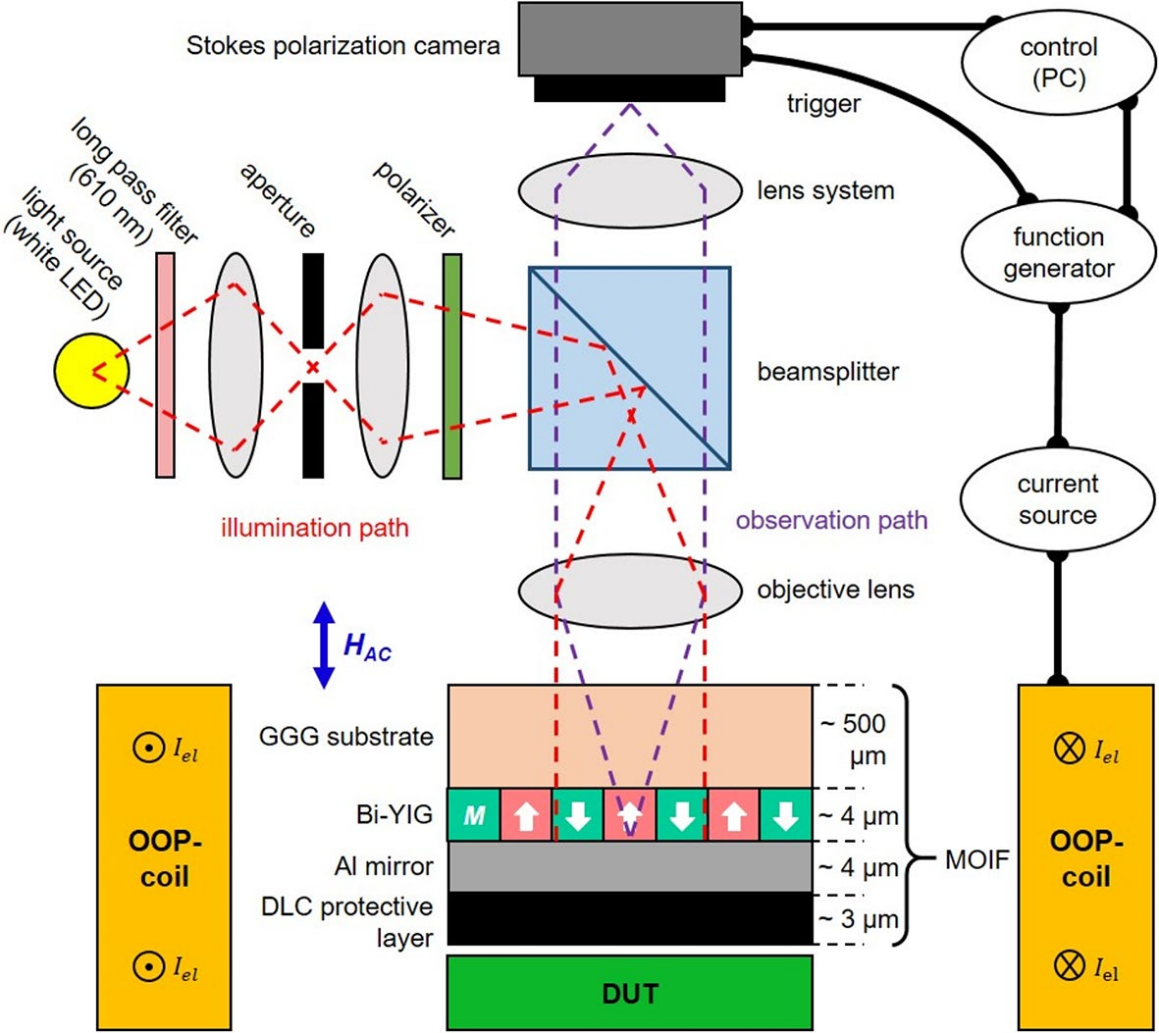
Schematic illustration of the sensor setup with simplified light path. A magnetooptical indicator film (MOIF) is used as the sensor head and placed on top of a device under test (DUT). The MOIF is separated from the DUT by a diamond like carbon (DLC) layer for protection and an aluminum mirror. The MOIF layer is observed using a conventional polarization microscope for high resolution. Linear polarized Köhler-type illumination at perpendicular incidence for polar MO contrast is realized using a white LED light source, a centrally positioned aperture and a subsequent polarizer. After traversing through the substrate and MOIF layer, the MOIF is imaged through an objective lens onto the primary image plane of the Stokes polarization camera. Polarization-retaining optics enable the camera to detect information about the polarization of the light, thereby determining the magnetization state of the sensor head. The function generator serves as a clock to synchronize the generated magnetic OOP AC magnetic field, with the camera exposure.

MOIFs utilize the magnetooptical Faraday effect. As linear polarized light passes through the magnetooptical layer, a rotation of the polarization axis ${\beta}_{\text{M}\text{O}}\left(x,y,T,H\right)$ depending on magnetization $\overrightarrow{M}\left(x,y,T,H\right)$ occurs. Typically, the backside of the MOIF is coated with a metallic mirror. This allows for indirect observation of the stray magnetic field from a sample or device under test (DUT) situated beneath the transparent magnetooptically active layer, as shown in Fig. 2. The light with the wave vector $\overrightarrow{k}$ is reflected at the mirror and passes through the MO active layer with thickness $d$ twice, resulting in a doubling of the Faraday rotation ${\beta}_{\text{M}\text{O}}$. The resulting spatial magnetooptical rotation can be described by^23^:

1 $${\beta}_{\text{M}\text{O}}\left(x,y,T,H\right) \:\approx\: V\left(\lambda,T\right)\cdot\:2d\cdot\:\left(\overrightarrow{M}\left(x,y,T,H\right)\cdot\:\overrightarrow{k}\right)$$

with $x$ and $y$ as the spatial coordinates. Thus, any change in magnetization through the magnetic field $H$ and temperature $T$ modulates the rotation of the polarization axis. Bismuth substituted rare earth yttrium iron garnets (Bi-YIG) are often used for magnetooptical sensor applications as they exhibit a large Verdet constant $V\left(\lambda,T\right)$ and thus a large Faraday rotation ${\beta}_{\text{M}\text{O}}$^24,25^.

MOIFs are generally used in reflection polarization microscopy in polar magnetooptical contrast configuration, which is sensitive only to the OOP component of magnetization^26^. The basics of such a setup have been implemented by a multitude of past works^6,10,12–16,27^. Using linearly polarized light and a subsequent analyzer, ${\beta}_{\text{M}\text{O}}\left(x,y,T,H\right)$ is converted into a change in light intensity $I\left(x,y,T,H\right)$. The measured intensity for each pixel of a camera is proportional to the original incoming intensity ${I}_{0}\left(x,y\right)$ and can be calculated using Malus’ law^20^:

2 $$I\left(x,y,T,H\right)={I}_{0}\left(x,y\right)\left[\left(1-\kappa\:\left(x,y\right)\right)\cdot{\text{sin}}^{2}\left({\alpha}_{0}+{\beta}_{\text{M}\text{O}}\left(x,y,T,H\right)\right)+\kappa\:\left(x,y\right)\right]$$

where $\kappa\:\left(x,y\right)$ is the polarization extinction coefficient. It depends on the polarizer-analyzer uncrossing angle ${\alpha}_{0}$. Consequently, the resulting camera image can be translated into information about the magnetic field and temperature if the relationship between these variables and magnetization is known.

Here, we demonstrate the use of garnet films with OOP anisotropy and large magnetooptical signal amplitudes for simultaneous field and temperature measurements based on a polarization sensitive camera. Sensing is achieved by evaluating the magnetooptical response of the MOIF at different points of the magnetization loop with constant magnetic field modulation.

## Results

### Quantification of Faraday rotation

Several methods for quantifiable magnetic imaging with MOIFs have been developed. Methods based on intensity calibration are susceptible to temporal drift effects, e.g. from changes in the illumination intensity ${I}_{0}$ of the light source. This can be overcome with the implementation of a beam splitter in the imaging path. It creates a secondary light beam or path which serves as a reference. Detection takes place by a photodetector for normalization^28^ or an additional camera to create a differential image^29^. Another approach uses a Faraday modulator in conjunction with a lock-in amplifier, requiring the scanning of a 2D map for imaging^29^. An alternative method uses multi-image polarimetry with different analyzer angles $\alpha$ to quantify the magnetooptical rotation. It enables the direct calculation of the angle of linear polarization (AOLP) of the incoming light using the ratio between the intensity of the images at different analyzer angles. This can be directly translated to the well-defined intensity independent magnetooptical parameter ${\beta}_{\text{M}\text{O}}$. This is accomplished either by rotating the analyzer and consecutive imaging^30,31^ or using the more recently emerged method via the utilization of a Stokes polarization camera^9^. The camera has four differently oriented polarizers in front of individual pixels arranged in a grid pattern^32^. The analyzer angles are 0°, 45°, 90° and 135°, enabling a direct calculation of the AOLP $\gamma$ incoming at the camera^33^:

3 $$\gamma\:\left(x,y,T,H\right)=\frac{1}{2}\text{Arg}\left[\left({I}_{0^\circ}\left(x,y,T,H\right)-{I}_{90^\circ}\left(x,y,T,H\right)\right)\right.\left.+i\left({I}_{45^\circ}\left(x,y,T,H\right)-{I}_{135^\circ}\left(x,y,T,H\right)\right)\right]$$

Using the offset $\alpha$ this can be directly translated into the magnetooptical Faraday rotation ${\beta}_{\text{M}\text{O}}$:

4 $${\beta}_{\text{M}\text{O}}\left(x,y,T,H\right)=\gamma\:\left(x,y,T,H\right)-\alpha$$

The general setup with a Stokes polarization camera is shown in Fig. 2. It enables repeatable quantitative information to be obtained with one single image frame. Thus, the time resolution is improved compared to multi-image systems utilizing a rotating analyzer or scanning methods. Furthermore, the quantitative imaging method eliminates the influence of optical defects and inhomogeneities in the illumination path, causing a spatially unevenly distributed light intensity ${I}_{0}\left(x,y\right)$, which cancels out in Eq. (3).

**Fig. 3:**
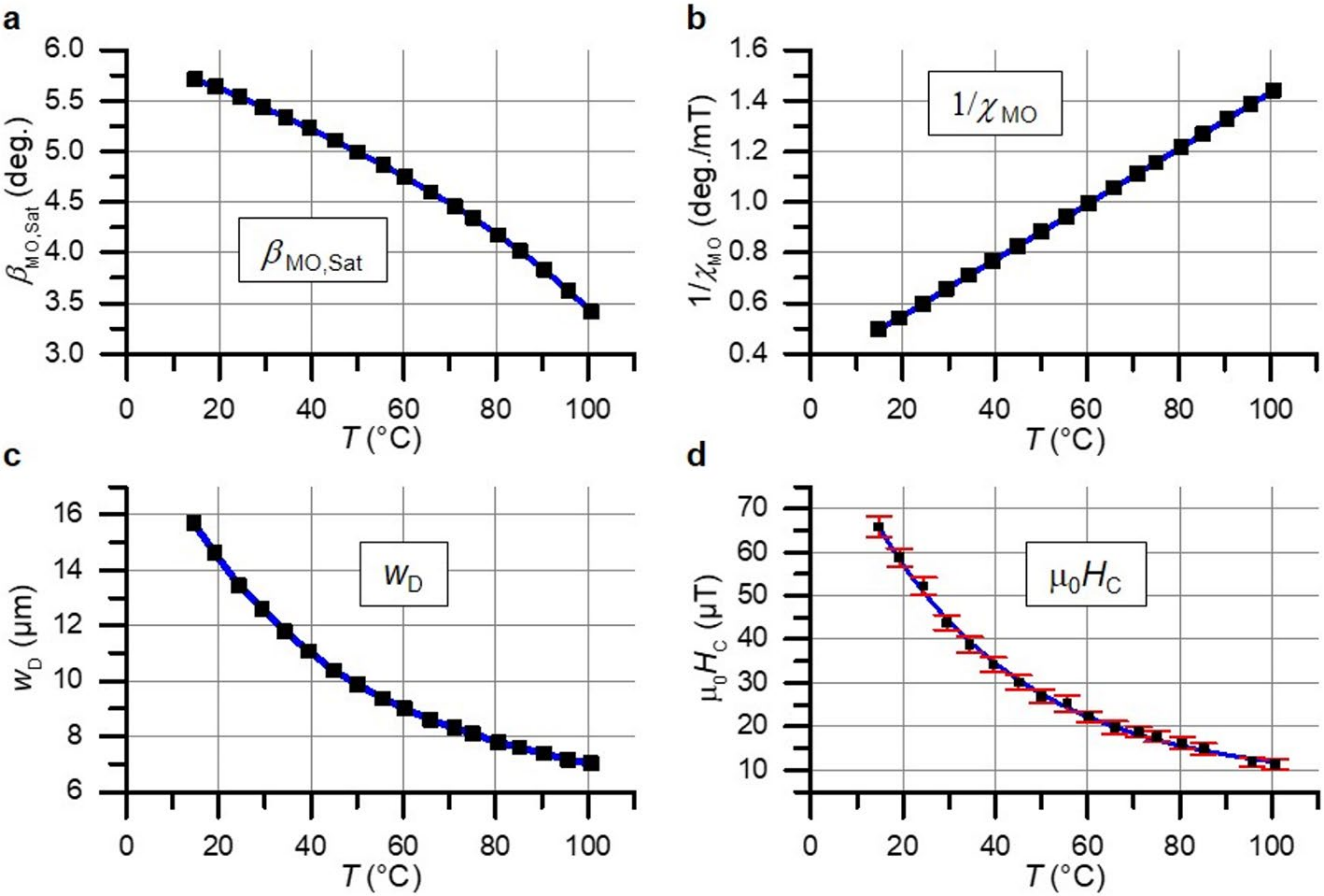
Temperature dependent quantitative parameters of the YIG sensor measured using a Stokes polarization camera. (**a**) Maximal magnetooptical Faraday rotation of polarization ${\beta}_{\text{M}\text{O},\text{s}\text{a}\text{t}}$ in OOP saturation. (**b**) Inverse of magnetooptical susceptibility ${\chi}_{\text{M}\text{O}}$ in the region of linear response for low applied fields. (**c**) Spatial parameter of magnetic domain period ${w}_{\text{D}}$ of the labyrinth domain structure. (**d**) Coercive field ${{{\upmu}}_{0}H}_{\text{C}}$of the hysteresis with its standard deviation from variations between different magnetization loops.

To independently extract values for magnetic field and temperature from the quantitative images of ${\beta}_{\text{M}\text{O}}$, temperature-dependent parameters shown in Fig. 3 are defined as mediators. The Faraday rotation of the garnet in saturation ${\beta}_{\text{M}\text{O},\text{s}\text{a}\text{t}}$ can be used to extract the temperature. The magnetooptical susceptibility ${\chi}_{\text{M}\text{O}}$ is defined as the response of ${\beta}_{\text{M}\text{O}}$ to externally applied fields in the linear region of the magnetization curve around zero field (see also Fig. 1):

5 $${\chi}_{\text{M}\text{O}}\left(T\right)=\frac{\delta\:{\beta}_{\text{M}\text{O}}\left(T,H=0\right)}{{\mu}_{0}\delta\:H}$$

It is approximated to be constant for small magnetic bias fields. The domain period ${w}_{\text{D}}$ is used to quantify the changes of the domain structure with temperature. See supplementary section S4 for details. It is also observed that the coercivity displays a characteristic change with temperature. Relying on magnetic domain wall motion, a notable disparity in coercivity between individual loops is observed.

### Sensor principle

To continuously extract information about these parameters, the magnetization of the MOIF is modulated by a sinusoidal external magnetic saturation field. This results in the MOIF to continuously traverse through a distinct magnetization loop, the characteristics of which dependent on both the magnetic bias field and temperature. Four specific images within one magnetization loop, two in saturation and two in the linear region close to zero field (Fig. 1a) are sufficient to extract data about the OOP magnetic field and the temperature.

The phase between exposure and modulation is chosen in such a way, that the average of ${\beta}_{\text{M}\text{O},1}$ and ${\beta}_{\text{M}\text{O},3}$ without an external field is close to zero. This maximizes the field range, in which the constant approximation of ${\chi}_{\text{M}\text{O}}$ is still valid. ${\beta}_{\text{M}\text{O},2}$ and ${\beta}_{\text{M}\text{O},4}$, obtained at magnetic saturation, are independent of any additional (small) magnetic bias fields. Their average, the offset of AOLP $\alpha$, is used for continuous axis zeroing for the Faraday rotation ${\beta}_{\text{M}\text{O}}$ shown in Eq. (4):

6 $$\alpha=\frac{{\gamma}_{2}+{\gamma}_{4}}{2}$$

While being primarily the constant angle between polarizer and camera analyzers, it corrects for quadratic magnetooptical effects^27,34^ not included in the model. From the difference of ${\beta}_{\text{M}\text{O},2}$ and ${\beta}_{\text{M}\text{O},4}$, a saturation ${\beta}_{\text{M}\text{O},\text{s}\text{a}\text{t}}$image is derived. Using a suitable fitting function $f$ based on Fig. 3a, the temperature is obtained:

7 $$T\left(x,y\right)=f\left[\frac{{\beta}_{\text{M}\text{O},2}(x,y)-{\beta}_{\text{M}\text{O},4}(x,y)}{2}\right]=f\left({\beta}_{\text{M}\text{O},\text{s}\text{a}\text{t}}\left(x,y\right)\right)$$

Any remaining slope of ${\beta}_{\text{M}\text{O},\text{S}\text{a}\text{t}}$ with field, e.g. from possible Faraday effects within the objective lens caused by the bias field, cancels out. At the selected magnetic field amplitude settings (Fig. 1a), for bias fields larger than approximately 1.2 mT saturation is not reached with certainty. This places an upper field limit on the measurements. The operational measuring range of temperature can be extended by increasing the amplitude of the modulation. Based on the temperature determined in Eq. (7), the magnetooptical susceptibility ${\chi}_{\text{M}\text{O}}$ can be calculated using the inverse relationship as shown in Fig. 3b with an empirical fitting function $g$:

8 $${\chi}_{\text{M}\text{O}}\left(T,x,y\right)=g\left(T\left(x,y\right)\right)$$

Together with the average of ${\beta}_{\text{M}\text{O},1}$ and ${\beta}_{\text{M}\text{O},2}$ the superimposed magnetic field offset ${\mu}_{0}H$ is obtained, assuming a linear relationship for small applied bias fields:

9 $${\mu}_{0}H\left(x,y\right)={\chi}_{\text{M}\text{O}}\left(T,x,y\right)\cdot\:\frac{{\beta}_{\text{M}\text{O},1}\left(T,x,y\right)+{\beta}_{\text{M}\text{O},3}\left(T,x,y\right)}{2}$$

The influence of the coercivity is canceled out as the measurement averages over both flanks of the hysteresis loop. For the used settings, the assumption of linearity starts to break down for magnetic OOP (bias) fields higher than approximately 1 mT. In general, the operational measuring range of the magnetic field can be extended by decreasing the modulation amplitude or image exposure. Due to the nonreproducibility of the magnetic domain structure, the resulting magnetic field image contains an additional noise signal that scales with the number of magnetic domains, respectively the domain period *w*_D_.

A Gaussian blur is used to eliminate the spatial magnetic domain signal. Acting as a low pass filter, it attenuates higher frequencies, including the majority of the domain signal, thereby limiting the spatial resolution to the domain period ${w}_{\text{D}}$. To select a suitable standard deviation and thus cutoff frequency for the Gaussian blur, ${w}_{\text{D}}$ is derived in parallel from the Fourier transformed images (Fig. 1c and e). The temperature dependency of *w*_D_ is shown in Fig. 3c. To reduce propagation of errors, the filter is also used prior to the calculation of the susceptibility from the temperature image using Eq. (8), given that the spatial resolution of field is already constrained. A comparable Fourier low-pass filter gives similar results. Without filtering the magnetic field images, the limit of spatial resolution of magnetic field with sufficient averaging is the optical resolution limit, which results from a combination of the diffraction limit, aberrations and the thickness of the Bi-YIG layer and is approximately 2.5 μm.

As all images are extracted individually for subsequent calculations, the temporal resolution of a continuous measurement is constrained by the frame rate of the camera. Given a frame rate of 56 Hz, this results in an acquisition rate of 14 Hz. For repeatable processes, exposures could be timed at the same phase, limiting temporal resolution to the exposure time.

### Sensor characteristics

**Fig. 4:**
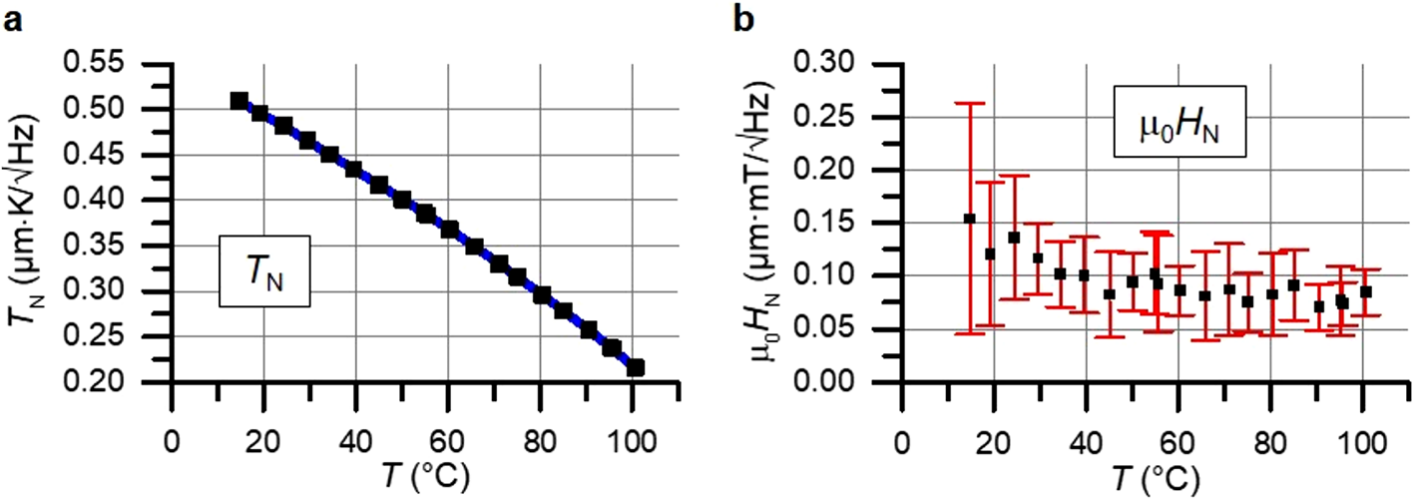
Sensitivity characteristics of the proposed sensor scheme. (**a**) Equivalent sensor noise level *T*_N_ of temperature and (**b**) magnetic field density µ_0_*H*_N_. The error bars represent the variation of noise between different sets of data. The noise is spatially and temporally normalized.

The equivalent noise of temperature (Fig. 4a) and magnetic field (Fig. 4b) are independent of the magnetic DC field and decrease with increasing temperature. For temperature, this correlation originates from the non-linear connection between ${\beta}_{\text{M}\text{O},\text{s}\text{a}\text{t}}$ to temperature. Therefore, the limiting factor of temperature noise can be attributed to the camera (or optical) system. This was confirmed by a comparison of detected ${\beta}_{\text{M}\text{O},\text{s}\text{a}\text{t}}$ noise with a plain mirror.

For the magnetic field measurements, the behavior correlates with both the nonreproducibility of coercivity and domain period for each magnetization loop (see also Fig. 3d). With increasing temperatures, the domain density increases, leading to a reduction in statistical errors. The contribution of other noise sources, e.g. from the camera system, is negligible for the given temperature range. At elevated temperatures, particularly when approaching the Néel temperature, the decrease of the magnetooptical susceptibility ${\chi}_{\text{M}\text{O}}$ and signal amplitude ${\beta}_{\text{M}\text{O},\text{s}\text{a}\text{t}}$ will result in the dominance of camera noise. Inhomogeneities of the Faraday rotation in saturation and magnetooptical susceptibility of the sensor are not corrected. They are significantly lower than the statistical noise.

**Fig. 5:**
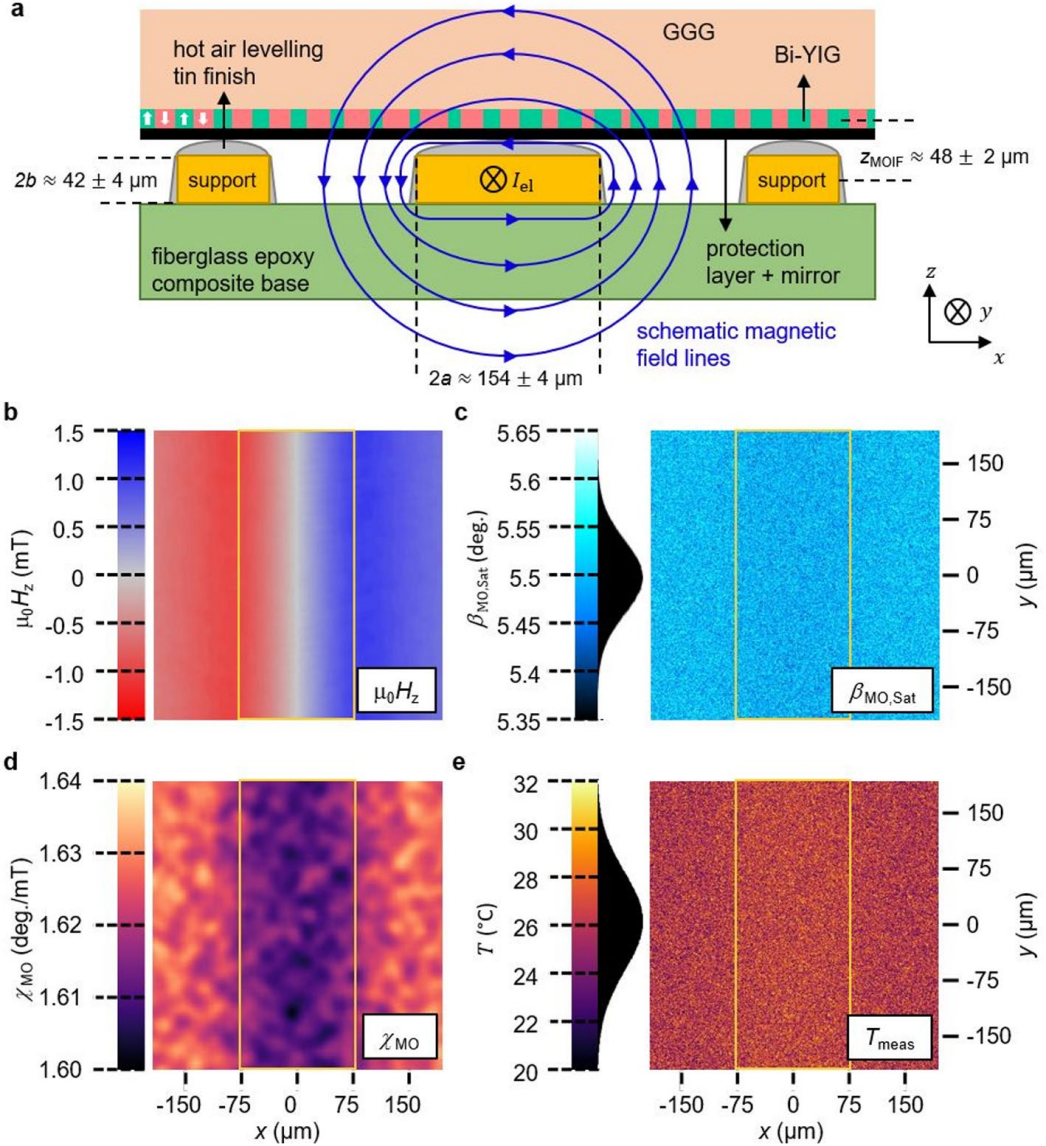
Schematic of the device under test with corresponding measured images at 0.6 A applied electrical current. (**a**) Sketch of the device under test with the MOIF on top including geometric parameters and schematic magnetic field lines caused by an electrical current ${I}_{\text{e}\text{l}}$ within a straight copper wire. Supports guarantee planar positioning of the MOIF. The measured images at thermal equilibrium with 0.6 A applied current are (**b**) magnetic OOP field distribution, (**c**) magnetooptical saturation magnetization map, (**d**) magnetooptical susceptibility map and (**e**) temperature map. The images are averaged over 14 acquisitions. (**b**) and (**c**) are filtered according to the domain signal. The contribution of the IP field and temperature is visible for the susceptibility. The position of the wire in the images is indicated with a yellow rectangle. The statistical distribution of the signal in (**c**) and (**e**) is indicated with a histogram.

### Sensor demonstration

A current-carrying straight cuboid copper wire of a custom printed circuit board^35^ is used to demonstrate the proposed method experimentally (Fig. 5a). The conductor has a slightly rough hot air levelling tin finish, resulting in a slightly trapezoidal shape with a convex top. The spatial dimensions of the wire $a$ and $b$ are extracted from a profile measurement with a confocal laser microscope. The illustration shows a rough estimation of the cross section. The resulting magnetic field image (Fig. 5b) is homogenous along the current direction. The image of ${\beta}_{\text{M}\text{O},\text{S}\text{a}\text{t}}$ (Fig. 5c) and thus temperature (Fig. 5e) is dominated by statistical noise, as the temperature is mostly homogenous in thermal equilibrium. A small influence and thus systematic error due to the magnetic IP field is expected as well. The effect of the magnetic IP field distribution is clearly visible in the filtered image of ${\chi}_{\text{M}\text{O}}$ (Fig. 5d).

An analytical Biot-Savart model is employed to calculate the expected magnetic OOP field distribution along the $y$-direction at the height of the MOIF. This model assumes a homogenous current density within the infinitely long wire and an infinitely thin MOIF^36^. The offset of magnetic field in the urban environment is measured and included in the model. Although the convex tin finish alters the assumed rectangular shape and introduces non-homogeneous current density due to the higher resistivity of tin compared to copper, the current predominantly flows through the approximately rectangular more conductive copper core. Consequently, the results are close to the original assumption. Additionally, this leads to some uncertain increase in the distance ${z}_{\text{M}\text{O}\text{I}\text{F}}$ between the MOIF and the actual conducting part of the wire due to the rough tin coating.

The calculated magnetic field distribution is also verified with an independent measurement using an established magnetic field imaging method utilizing an MOIF with IP anisotropy^10,12,15,21^. The distance ${z}_{\text{M}\text{O}\text{I}\text{F}}$ is calculated using multiple fits of the Biot-Savart model with this data at different known applied currents and is repeatable between experiments. The data of the verification measurement coincides with the calculation within an appropriate error tolerance.

**Fig. 6:**
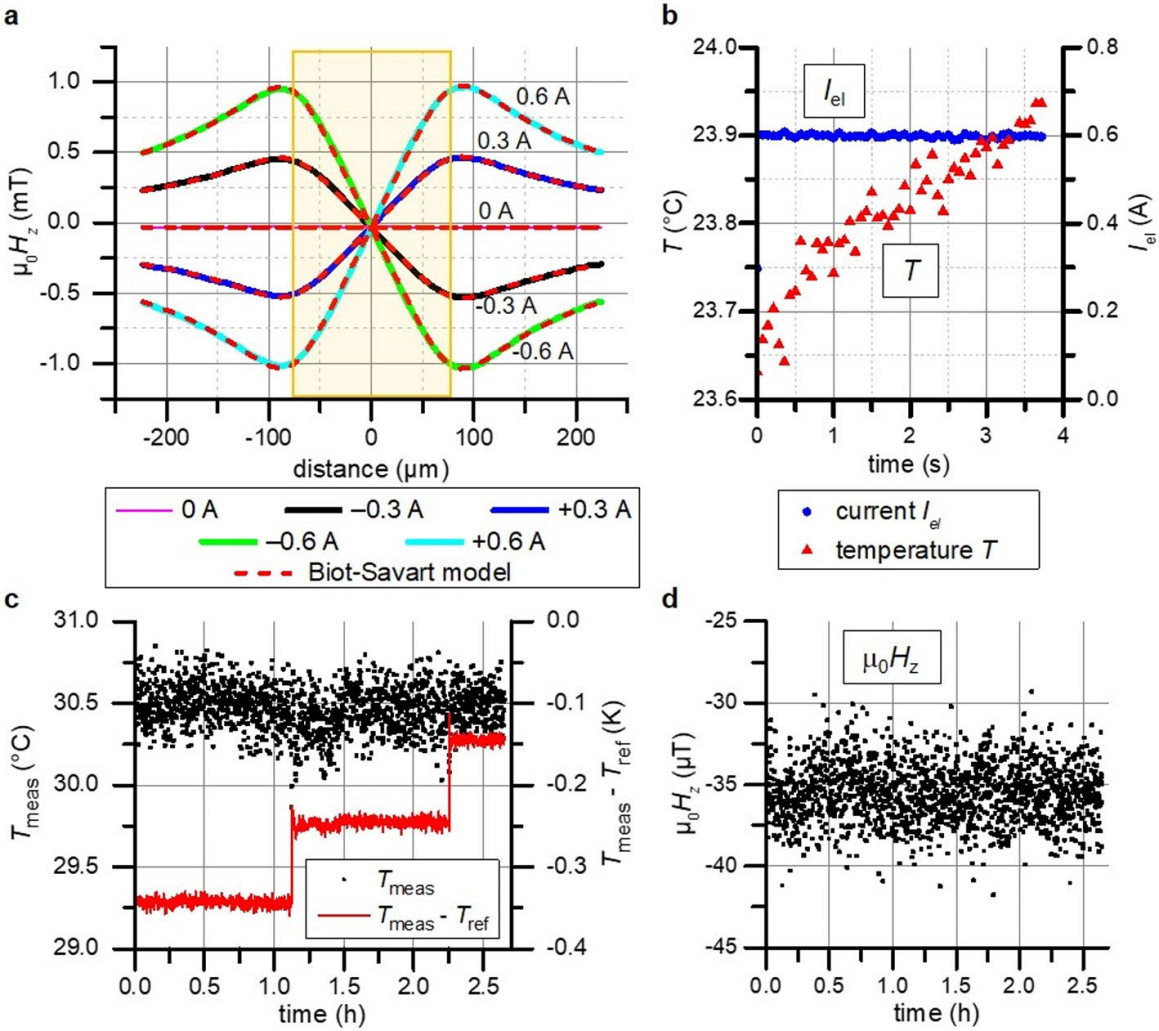
Experimental demonstration of the simultaneous derivation of magnetic field distribution, temperature and electrical current. (**a**) Derived OOP magnetic field distribution from a line profile along the $x$-direction. The position of the conductor is indicated in yellow. The experimental results are averaged over 14 acquisitions across a 200 μm wide region over the conducting wire. The results from an analytical Biot-Savart model are superimposed for comparison. The average temperature at thermal equilibrium is measured for 0.3 A to be 24.2 °C and for 0.6 A measured to be 26.1 °C. (**b**) Evolution of measured temperature and current after applying a current of 0.6 A with the onset of current at 0 s. No temporal averaging was applied. Simultaneous long-term stability measurement of (**c**) temperature and (d) a constant magnetic field. The magnetooptically measured temperature *T*_meas_ is compared to the reference measurement using a thermocouple *T*_ref_ during a step wise increase.

The magnetic field distribution measured by the proposed method is measured at thermal equilibrium for varying applied currents (Fig. 6a) and compared to the analytical model. The calculated data nearly exactly matches the experimentally determined magnetic field distribution. The additionally measured urban DC offset field corresponds to the independently measured value using a Hall probe. Observed small deviations are within the expected range of variation due to measurement and urban noise. Minimal differences are contributed to limitations of the used model and the influence of the additionally acting IP field contribution, which slightly change the measured saturation and thus susceptibility.

Using the magnetic field distribution in Fig. 6a, it should also be possible to deduce information about the width parameter of the wire $a$ through fitting. Small wires can be distinguished by differences in the amplitude of the peaks, while larger wires could be differentiated by the position of the peaks along the $x$-axis. In this demonstration, such fits provide comparable results on conductor width as the confocal microscope measurement.

Using an electrical current calibration based on the analytical model in Fig. 6a, the total current and temperature are measured in the period immediately after the current is applied (Fig. 6b), demonstrating temporal resolution of a continuous parallel measurement of current and temperature. The stability of temperature (Fig. 6c) and magnetic field measurement (Fig. 6d) is validated by long-term monitoring over several hours. The stepwise increase in temperature does not appear to exert any influence on the measurements. The observed slight differences between reference and measurement temperature can be attributed to temperature differences between the reference and the sensor garnet, as well as an error due to the used empirically fitting function $f$ from Eq. (7). No statistically significant drift is observed in either the measured magnetic field or the derived temperature.

## Discussion

In conclusion, the utilization of an MOIF with OOP anisotropy as a magnetooptical sensor element for quasi-parallel quantitative imaging of magnetic field, as well as measurement of temperature and electrical current in circuitry is demonstrated. For this purpose, the magnetooptical saturation and offset signal are acquired using a triggered image acquisition at four characteristic points during a saturating magnetization loop. Subsequent characterization reveals that the equivalent noise levels of both temperature and magnetic field decrease with increasing temperature. The overall limiting factor for the temperature measurement is the noise of the camera. The magnetic field measurement it is limited by the nonreproducibility of coercivity due to magnetic domain effects. A noise level of magnetic field of up to 0.15 µT/√Hz has been achieved parallel to a temperature noise level of up to 0.4 mK/√Hz.

The presented technique offers a practical method for spatially resolved measurement of magnetic fields of up to 1.0 mT in environments with varying temperatures, facilitating the characterization of micrometer-sized electronic devices and materials by means of optics. The method allows for fast mapping of magnetic field over large areas and reaches a comparable sensitivity to scanning Hall microscopy^7^. Realization of current distribution imaging based on magnetic field images using established mathematical algorithms^37^ with simultaneous temperature imaging of the sensor should be possible.

## Materials and methods

### Sensor material

The used sensor garnet is a commercially available magnetooptical sensor from matesy GmbH^38^. The MOIF consists of a 3 μm thick bismuth substituted rare earth yttrium iron garnet layer epitaxially grown on a gallium gadolinium garnet (GGG) with OOP anisotropy^25,39^. It is designated as product type A and shows a Néel temperature of 138 °C.

### Imaging system

The basis of the experimental sensor setup is a magnetooptical widefield microscope^27^ (Fig. 2). For all experiments, an objective with a numerical aperture of 0.25 is used. The total field of view of the images is 458 μm times 548 μm. An exposure time of 3.3 ms is set as a compromise between measuring range and noise. To minimize wavelength dependent distortions in the measurement, a 610 nm long-pass filter is placed after the white LED light source. The illumination aperture in the back focal plane of the magnetooptical microscope is centralized for polar magnetooptical sensitivity. The optical diffraction limit is 2.4 μm.

### Stokes polarization camera

The camera used in this paper is a DZK 33UX250 (The Imaging Source) using a Sony CMOS Pregius Polarsens IMX250MZR Sensor chip with a 3.45 μm pixel size^40^. A bilinear interpolation is performed for each of the individual intensity images of the four analyzer angles at 0°, 45°, 90° and 135° of the camera. To correct for static differences in sensitivity and the individual analyzer angles, the camera has been calibrated within the optical system. The angle between the polarizer and the camera is optimized for minimal noise in ${\beta}_{\text{M}\text{O}}$. For details see supplementary section S1-S3.

### Noise characterization

A statistical analysis of images taken at different temperatures and external applied bias fields is performed. The standard deviation between the measurements is considered as the sensor accuracy. The resulting noise of temperature and magnetic field is normalized spatially for one µm^2^ and temporally for one second of total exposure time. Dominating white noise is assumed for the analysis.

### Magnetic field and temperature control

For magnetic field modulations, a Helmholtz-coil is used to ensure homogeneity of any applied fields. The sensitivity measurements are done in thermal equilibrium. The temperature is variated using a Peltier-element. To reduce effects of magnetic fields generated by the element, it is spatially separated from the sample and thermally connected via a block of copper plates. A reference temperature *T*_ref_ is measured using a thermocouple placed in the block. The long-term measurements use an active PID control of temperature.

## Electronic supplementary material

Below is the link to the electronic supplementary material.

Supplementary Material 1

## Data Availability

The datasets generated and analyzed during the current study are available from the corresponding author on reasonable request.
