# Supplementary material for: Quantitative magnetooptical analysis using indicator films for the detection of magnetic field distributions, temperature, and electrical currents
Source: Sci Rep. 2024 Oct 26;14:25459. doi: 10.1038/s41598-024-74684-y (PMC11513121; doi:10.1038/s41598-024-74684-y)
Supplement: Supplementary file 1 — Supplementary Material 1 [file 41598_2024_74684_MOESM1_ESM.pdf]

Supplementary

Quantitative magnetooptical analysis using indicator films for the detection of magnetic field distributions, temperature, and electrical currents

Michael P. Path[1]*, Jeffrey McCord[1,2]

[1]Nanoscale Magnetic Materials - Magnetic Domains, Department of Materials Science, Faculty of Engineering, Kiel University, 24143, Kiel, Germany

[2]Kiel Nano, Surface and Interface Science (KiNSIS), Kiel University, 24118 Kiel, Germany

S1. Stokes camera interpolation

As each image of one of the four individual analyzer angles of the Stokes camera is undersampled, an interpolation is necessary to reconstruct a full image at the original resolution. Therefore, a bilinear interpolation algorithm is used to calculate a 2448×2048 full-resolution image of each analyzer angle 1224×1024 image of the chip according to the analyzer pattern placed on the sensor chip (Figure S1). Then, these images are used to calculate the β_{MO} image at original resolution. For the statistical analysis, the number of non-interpolated pixels is used.

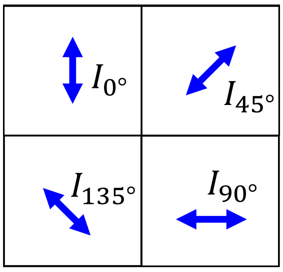

Figure S1: Unit cell of the analyzer pattern of the Stokes camera CMOS sensor chip.

S2. Stokes camera uncrossing angle optimization

To find the ideal angle between polarizer and the Stokes-camera, images of a plain mirror are taken and the standard deviation of AOLP within those images is calculated. Then, the camera is rotated until a minimum of noise of β_{MO} is reached. This means that noise of the intensities of the different pixel types has the least influence on the β_{MO} at this angle.

S3. Stokes camera calibration procedure

Due to imperfections of the analyzer angles and sensitivity differences of the individual pixels, a calibration is necessary[32]. As the deviations between AOLP and the angle set by the polarizer are expected to be small and assuming only small variations of the individual analyzer angles from their idealized values, their influence on the measured AOLP can be estimated as linear. Consequently, as the properties of the individual pixels stay constant, the differences in pixel sensitivity and analyzer angle will approximately convert to a constant offset of the locally measured AOLP. To correct for this systematic error, every pixel of the acquired AOLP image is divided by a corresponding value of a calibration image. This calibration image is calculated from

an image which is slightly out-of-focus with high averaging using the same microscopic setup as the actual experiment. This image is then normalized via division of its average value of AOLP. The functionality of this method is proven empirically, as an average reduction of more than 99% of the standard deviation of a plain mirror image is measured. For an image of the saturated sensor garnet, the measured reduction is at 92%. The method is less effective for the garnet in saturation, as the non-linearity of the AOLP calculation take maximal effect due to the magnetooptical rotation in this case. A calibration using a flat subtraction instead of a division of the calibration image achieves a similar result. The same calibration image is used for all presented data.

S4. Domain period

The domain period is determined from the spatial frequency of the peak location of the largest peak in the radial profile of the Fourier transform of a domain image averaged over 3.3 ms around zero applied field during a 14 Hz sinusoidal magnetic field excitation with 6 mT amplitude.

The alternative of a temperature measurement using the domain period has been investigated. While such a method is found to be possible in principle, several problems have been identified. In addition to temperature, the domain period has additional dependencies on IP field, frequency of excitation, history and local defect density. A large field of view is necessary for the 2D Fourier analysis to obtain repeatable results. For images taken with 0.05 s exposure time during a 200 Hz ramp modulation with 8 mT peak-peak amplitude using the same imaging system and utilizing the whole field of view, a temperature independent temperature sensitivity of 60 mK/√Hz is obtained, which is several orders of magnitude lower than the presented results obtained via the change of saturation magnetization.

S5. Sensor characterization

To characterize the sensing principle, a statistical analysis of images taken at different temperatures and external applied bias fields was performed. For the equivalent temperature noise level (Figure 3a), the standard deviation between the temperature values of all non-interpolated pixels within the image is used. The equivalent magnetic field noise (Figure 3b) is measured as the standard deviation between different acquisitions at a single temperature, where the resulting noise levels are normalized with respect to the square root of the total time of exposure. This assumes dominating white noise. The temperature noise is also normalized for one µm^2 to characterize spatial accuracy. This assumes white noise along the spatial dimensions. A Fourier analysis shows this to be approximately true.

The equivalent noises are independent of the magnetic DC field and decrease with increasing temperature. For temperature, this correlation originates from the non-linear connection between $\beta_{\mathrm{MO,sat}}$ to temperature. This is indicated by the linear relationship between $\delta T/\beta_{\mathrm{MO,sat}}$ and the temperature noise in Figure S2a. Since the camera noise remains constant with temperature, the

limiting factor of temperature noise can be attributed to the camera (or optical) system. This is confirmed by a comparison of detected $\beta_{\mathrm{MO,sat}}$ noise with a plain mirror.

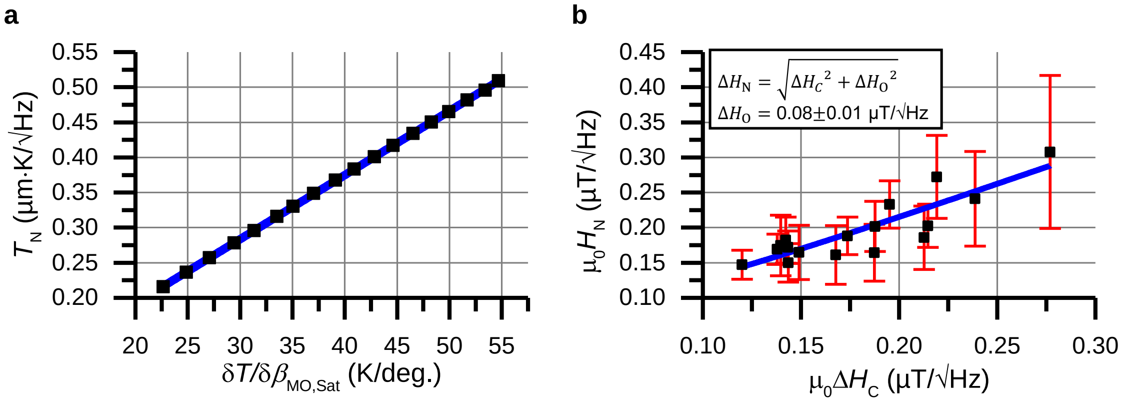

Figure S2: a) Temperature noise with a gradient of Faraday rotation at saturation magnetization $\delta\beta_{\mathrm{MO,sat}}/\delta T$ to temperature. b) Magnetic field noise over the deviation of the coercive field $\mu_0\Delta H_{\mathrm{C}}$ normalized over time. The offset $\mu_0\Delta H_{\mathrm{O}}$ originates from the significantly lower effective field noise contribution from other sources. ΔH_{O} is assumed constant.

For the magnetic field measurements, the behavior correlates with both the coercivity and the domain period, as they follow a similar decay with temperature. Thus, the dominating part of noise is magnetic in nature. It originates from the variations in between each magnetization loop. The coercivity H_{C} differs between each loop on average about ΔH_{C} (Figure 2d) due to irreversible domain effects. This directly translates into an error in the measured magnetic field (Figure S2b). The shift in the domain wall energy with changing OOP anisotropy is an influencing factor on temperature dependent behavior. Furthermore, more domains are present within the images for a smaller domain period. Thus, the statistical error is reduced for higher temperatures. In comparison, the contribution of other noise sources, e.g. from the camera system or urban noise ΔH_{O} (Figure S2b), is negligible for the given temperature range. Nonetheless, for temperatures close to the Néel temperature, this part might dominate as the camera noise increases as the magnetooptical susceptibility and the signal amplitude decreases.

S6. Magnetic field and current calculation

The used analytical formula is derived from the Biot-Savart law for the out-of-plane part of magnetic stray field H_{z} caused by an electrical current I_{el} in a cuboid conductor. The magnetooptically active layer is approximated as infinitely flat and the current density homogenous. The corresponding spatial parameters are depicted in Figure 5a [35]:

$$H_{\mathrm{z}}(I_{\mathrm{el}}, x, z) = \frac{I_{\mathrm{el}}}{8\,\pi ab} \cdot \left\{ \begin{matrix} g \cdot \left[\frac{1}{2}\ln\left(\frac{u^2+g^2}{v^2+g^2}\right) + \frac{u}{g}\arctan\left(\frac{g}{u}\right) - \frac{v}{g}\arctan\left(\frac{g}{v}\right)\right] \\ -h \cdot \left[\frac{1}{2}\ln\left(\frac{u^2+h^2}{v^2+h^2}\right) + \frac{u}{h}\arctan\left(\frac{h}{u}\right) - \frac{v}{h}\arctan\left(\frac{h}{v}\right)\right] \end{matrix} \right\} + H_{\mathrm{Off}}$$

With

$$u = a - x - x_0$$

$$v = -a - x - x_0$$

$$h = -b - z$$

$$g = b - z$$

The dimensions of the wire a and b are determined using a confocal microscope. The position of the wire x_0 and the distance of the garnet to the wire z_{MOIF} are determined using multiple fits of the Biot-Savart law to the measured magnetic field distribution for different applied currents. This is independently measured to the proposed method using an MOIF with IP-anisotropy[10,12,15,21]. Using a low superimposing IP-field, the IP-MOIF is driven into a single-domain state. Then, using the estimate from the confocal measurement, a fit with a known current is done to check for the geometry parameters. The result of the width coincides (with a given margin of error) with the confocal measurement.

The result of x_0 is used as the zero-point of the x-axis in all depictions. The obtained values of the DC offset and the temperature are in supplementary table 1.

Supplementary table 1: Measured temperature and magnetic OOP bias field in the demonstration of spatial field resolution.

applied current I_{el} in A	field offset $\mu_0 H_{\mathrm{Off}}$ [µT]*	temperature in °C
-0.6	-29.3	26.09
-0.3	-29.6	24.24
0.3	-35.0	24.26
0.6	-34.4	26.13

*Measured $\mu_0 H_{\mathrm{Off}}$ with a Hall probe: -35 $\pm$ 5 µT; variations due to urban noise expected

The measured current shown in Figure 6b is acquired from the magnetic field difference between the peaks, using the average of 11 µm wide region which are 91 µm away from the centre of the conductor. The numerical integral of the model of the same regions is used as a calibration for the current measurement and is calculated to be 1.185 A/mT.

S7. Deduction of wire geometry from the measured magnetic field distribution

Assuming a rectangular shape, information about the size of the wire can be obtained using a fit with the sought parameter as the free variable. In case of the wire width parameter *a*, its influence is shown in figure S3 for given equivalent to the shown demonstration. As the amplitude of the magnetic field in the peaks changes, the ability to distinguish a larger and a smaller wire is limited by the optical resolution. Variations of the wire thickness b, if b is significantly smaller than the distance to the sensing plane z, have a negligible influence on the magnetic field distribution.

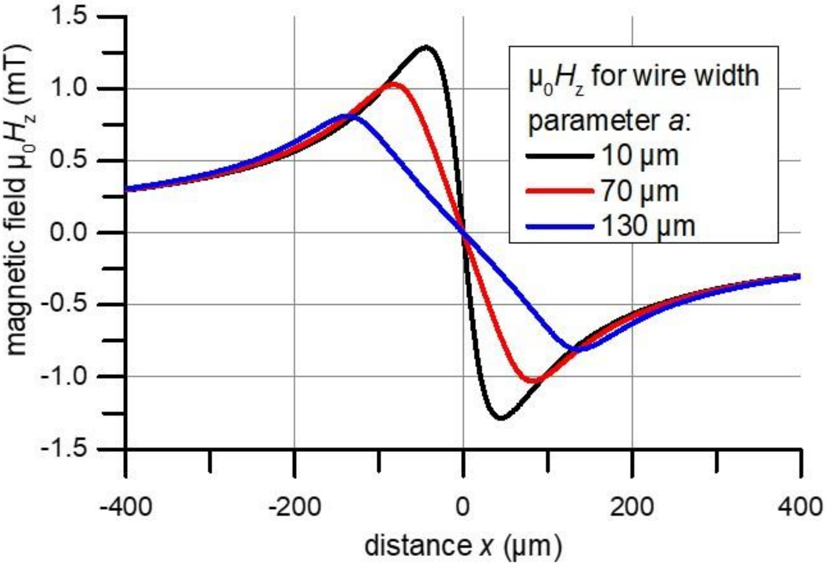

Figure S3. Calculated magnetic field distribution for different widths of the wire with I = 0.6 A, z_{MOIF} = 48 µm and b = 21 µm.
